# Masculinity norms and help-seeking intentions after experiences of interpersonal violence

**DOI:** 10.1093/eurpub/ckac131.332

**Published:** 2022-10-25

**Authors:** N Komlenac, E Lamp, F Maresch, M Hochleitner

**Affiliations:** Institute of Diversity in Medicine, Medical University of Innsbruck, Innsbruck, Austria

## Abstract

**Background:**

High levels of self-compassion might increase men’s willingness to seek formal help, including those men, who rigidly conform to masculinity norms (CMN). However, self-compassion has rarely been considered as an enabling factor for women’s help-seeking intentions. The current study analyzed the links between CMN, self-stigma, self-compassion and willingness to seek formal help after experiences of interpersonal violence (IPV) in women and men.

**Methods:**

A cross-sectional online-questionnaire study was conducted with 491 German-speaking participants (65.6% women/34.4% men; age: M = 36.1 years; SD = 14.2). Participants read three vignettes about experienced IPV. Afterwards, they indicated how likely they would be to seek medical or psychological help if they were in the main characters’ situation. The Conformity to Masculine Norms Inventory, Self-Stigma of Seeking Help Scale, Self-Compassion Scale were used. Separate manifest path models were calculated for women and men.

**Results:**

CMN and low self-compassion were linked to strong self-stigma. In turn, strong self-stigma was associated with reduced help-seeking intentions. In men, the interaction term Self-Compassion x CMN on self-stigma indicated that strong CMN was linked to increased self-stigma, especially in men with low self-compassion. Also, indirect links between CMN and help-seeking intentions via self-stigma have been found. However, in people with strong self-compassion, direct links between CMN and help-seeking intentions became evident.

**Conclusions:**

In women and men self-compassion was linked to reduced self-stigma. As is suggested from past research, self-compassion might “buffer” the link between CMN and self-stigma, especially in men. However, there might be an influence of strong CMN on help-seeking intentions also in people with strong self-compassion. This route might not be mediated by self-stigma and therefore, future research is needed to detect other potential mediators.

**Key messages:**

• Self-compassion might “buffer” the link between CMN and self-stigma in men.

• Future research is needed to study mediators between strong CMN and help-seeking intentions in people with strong self-compassion.

